# Toxicity Evaluation of Selected Plant Water Extracts on a Honey Bee (*Apis mellifera* L.) Larvae Model

**DOI:** 10.3390/ani12020178

**Published:** 2022-01-12

**Authors:** Roksana Kruszakin, Paweł Migdal

**Affiliations:** Department of Environment, Hygiene and Animal Welfare, Wroclaw University of Environmental and Life Sciences, 51-630 Wroclaw, Poland; pawel.migdal@upwr.edu.pl

**Keywords:** honey bee, in vitro larvae rearing, water plant infusions, larval diets, coriander, tansy, greater celandine

## Abstract

**Simple Summary:**

The honey bee is a very important link in food production. Conducting research into the influence of various factors on its biology is a very important element in the preservation of its population and pollination potential. It is very common for beekeepers to use plant extracts to combat bee pathogens. However, there are few studies assessing the effect of these extracts on the individual development stages of worker bees. Our research has shown that this is an essential element when making a decision on the use of a given extract, as it could negatively affect the survival of bee larvae. The proper development of worker bee larvae ensures an appropriate number of pollinators in the environment and the survival of bee colonies.

**Abstract:**

So far, larval rearing in vitro has been an important method in the assessment of bee toxicology, particularly in pesticide risk assessment. However, natural products are increasingly used to control honey bee pathogens or to enhance bee immunity, but their effects on honey bee larvae are mostly unknown. In this study, laboratory studies were conducted to determine the effects of including selected aqueous plant infusions in the diet of honey bee (*Apis mellifera* L.) larvae in vitro. The toxicity of infusions from three different plant species considered to be medicinal plants was evaluated: tansy (*Tanacetum vulgare* L.), greater celandine (*Chelidonium majus* L.), and coriander (*Coriandrum sativum* L.). The impact of each on the survival of the larvae of honey bees was also evaluated. One-day-old larvae were fed a basal diet consisting of distilled water, sugars (glucose and fructose), yeast extract, and freeze-dried royal jelly or test diets in which distilled water was replaced by plant infusions. The proportion of the diet components was adjusted to the age of the larvae. The larvae were fed twice a day. The experiment lasted seven days. Significant statistical differences in survival rates were found between groups of larvae (exposed or not to the infusions of tansy, greater celandine, and coriander). A significant decrease (*p* < 0.05) in the survival rate was observed in the group with the addition of a coriander herb infusion compared to the control. These results indicate that plant extracts intended to be used in beekeeping should be tested on all development stages of honey bees.

## 1. Introduction

Honey bee (*Apis mellifera* L.) colonies are exposed to natural plant compounds, microorganisms, pesticides, environmental pollutants, and all kinds of drugs used by beekeepers every season [1]. During foraging, workers may meet a whole range of different biologically active substances, including those with toxic effects, which are then transferred to the hive and stored [2]. Studies conducted by American scientists have confirmed the presence of 121 different pesticides and metabolites in wax and pollen samples, several of which are compounds used by beekeepers to control colony pests [3,4]. For this reason, many beekeepers, despite having access to commercially used, synthetic protection agents, decide to use their natural substitutes, usually in the form of plant infusions or water extracts, which are easy and cheap to prepare [3].

The growing interest in the use of plant extracts in the treatment of human and animal diseases contributes to the expansion of the currently available knowledge on the use of plant products in medicine, animal production, and agriculture [5]. However, using natural substitutes for synthetic drugs also carries risks. If not properly selected and prepared, they can exhibit significant toxicity [6]. Therefore, it has become an interesting topic to investigate the effects that plants used as natural repellents of invertebrate organisms may have on the honey bee, starting from the earliest developmental stage—the larvae [7]. In addition, the development of existing techniques used for in vitro larval culture enables new modifications and improvements to existing culture protocols using different compositions and nutritional additives [6].

The biological activity of various plant species has been demonstrated against bee diseases such as American foulbrood, varroosis and nosemosis [8]. An example of this is the use of tansy (*Tanacetum vulgare* L.) herb and flower baskets as an agent against the parasitic mite *Varroa destructor*. The effectiveness of this method has been confirmed—the smoke from the common marigold caused a 70–90% decrease in the mite. The dried above-ground plant parts are burned in a vacuum cleaner and the interior of the hive is fumigated [9]. In addition, beekeeping practices have been described that suggest that, to attract bees to their hives, beekeepers use an aqueous solution which is a mixture of extracts from three plant species: tansy, spike lavender (*Lavandula latifolia* Medik.), and melissa (*Melissa officinalis* L.) [10]. On the other hand, in a study on the toxicity of polyphenols contained in coriander (*Coriandrum sativum* L.) on larvae of the greater wax moth (*Galleria mellonella* L.), a pest that feeds on beeswax, it was proven that the exposure of larvae to the above-mentioned compounds caused high mortality, especially for a high dose (30 µL/mL), which led to 100% mortality within four days [11].

So far, research on the potential use of herbal plants in beekeeping has focused on attempts to use them to combat specific diseases by exploiting their antibacterial, antifungal, or antiviral properties [12]. There have been very many experiments testing the effects of pesticides and plant extracts on adult bees, but there are few studies assessing the effects of the compounds they contain on the earlier life stages, including larvae [7]. This is why the breeding of honey bee larvae under laboratory conditions is such an important tool, especially the most numerous worker bees responsible for the proper functioning of a bee colony, as this allows for reproducible and standardized results [13].

The aim of this study was to compare the survival rate of honey bee larvae exposed to biologically active compounds contained in the aqueous infusions of three plant species used in herbal medicine: tansy (*T. vulgare* L.), greater celandine (*C. majus* L.), and coriander (*C. sativum* L.).

## 2. Materials and Methods

With the hives empty, labeled bee combs were placed in isolators. A honey bee queen was confined to an empty comb for 24 h to obtain larvae of defined age. After 24 h, it was confirmed that the queen had laid eggs in the cells. The age of the eggs after the release of the queen was estimated at 12 ± 12 h. After 3 days, combs with developing broods were transported to the laboratory under stable temperature and humidity conditions (34 ± 1 °C, 80–90% humidity). Frames with 1-day-old larvae of the honey bee were obtained from the didactic and scientific apiary of the Wrocław University of Environmental and Life Sciences.

The larvae were transferred to plastic cage bowls embedded in a 48-well tissue culture plate. A dose of food (diet A) equal to 20 µL was dispensed into the bottom of the bowl using a pipette. Using a metal larval transfer spoon, larvae were transferred, one per cell, at the L1 stage from the bee frame. The transfer of damaged larvae and those that were not transferred in the first attempt was avoided. Dead, defective, or questionable larvae were replaced. It took no more than 10 min to fill each plate. Optimal temperature (34 ± 1 °C) and humidity (80–90%) were maintained in the room to prevent the larvae from drying out.

In the whole experiment, 768 larvae were used. There were 3 experimental groups and 1 control group, 4 repetitions, with48 larvae in each repetition, and a total of 192 larvae for each group. The remaining larvae in the patch were deposited in the bee colony.

### 2.1. Diet

The diet for rearing worker bees consisted of distilled water, sugars (fructose and glucose), yeast extract, and royal jelly, all in powder form. Freeze-dried royal jelly was used (300 mg of freeze-dried royal jelly corresponds to 900 mg of fresh royal jelly).

In the experiment, a diet consisting of 3 compositions (A, B, and C) was prepared for the control group and fed to the larvae according to the requirements of their developmental stage for a period of six days. Diets A, B, and C differed in the proportion of components they contained (Table 1) [11]. In the study groups, distilled water was replaced with water extracts from the tansy, greater celandine, or coriander. Other components of the diet were unchanged [12].

Each portion of the diet was heated to 34.5 °C before each feeding. Diet A was given only on the day of larval transposition (D0) and was the only one not divided into two doses, diet B was given on day D2 (second day after transfer), and diet C on days D3, D4, and D5. There were a total of five feedings over a six-day period, with no feeding occurring on the second day of rearing (Table 2). The larvae were fed twice a day (to avoid waterlogging of the larvae when the entire diet volume was added at once) from day three [11].

Plates with translated larvae were placed in an incubator for 7 days at 34.5 °C and a relative humidity of at least 90 ± 3%.

### 2.2. Viability Assessment of Bee Larvae

Observations to assess larval survival were conducted for 6 consecutive days from the day of larval transfer. A larva was defined as alive when it retained a turgor, a characteristic pearly sheen could be observed, and respiratory fistulae moved. A larva was classified as dead when its color turned brownish and much duller, swelling appeared, and it remained motionless or became waterlogged.

### 2.3. Water Plant Infusions

The plant materials were collected from their natural sites, i.e., far from expressways, meadows and forests far from expressways. The exception was coriander, which was obtained commercially. The green parts of the plants were cleaned under a stream of running water and allowed to dry. Only the leaves and stems were used. Finely chopped plant fragments (5.00 ± 0.1 g) were placed in a beaker filled with 1000 mL of water at 80 °C. This was covered for 15 min. Using two sieves with different mesh diameters, plant fragments were separated from the finished infusion.

### 2.4. Statistical Analysis

Data analysis was performed using the statistical package R Survival in R Studio version 3.4.4. The survival function was calculated using the Kaplan–Meyer estimator. The significance of differences between experimental groups was estimated by a log-rank test and the Cox regression model. *p* ≤ 0.05 was considered statistically significant.

## 3. Results

A total of 221 larvae died during the experiment: 74 fell in the coriander group; 51 in the greater celandine group; 41 in the tansy group. In the control group, 55 dead larvae were recorded, which was 28.6% of the initial population. The highest survival values were observed in trials with the addition of an aqueous extract of tansy. The probability of survival in this group over the course of the experiment was 78.6%. A slightly lower percentage was recorded in the case of greater celandine: 73.4%. The highest mortality occurred in the group exposed to compounds contained in coriander.

**Figure 1:**
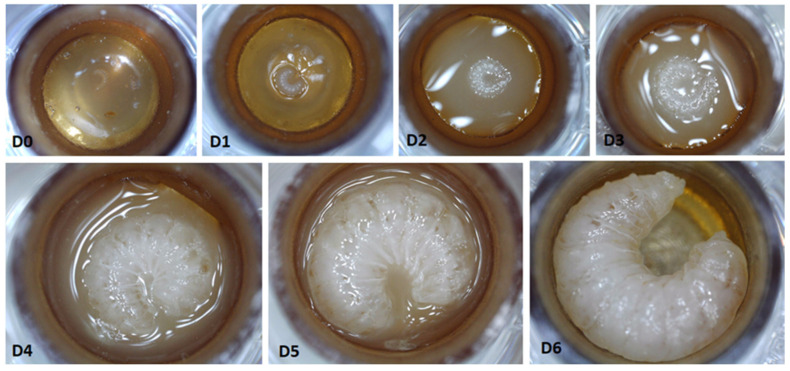
Development of a larva from day of translation (**D0**), and one day (**D1**), two days (**D2**), three days (**D3**), four days (**D4**), five days (**D5**), and six days (**D6**) after translation. The larva on day six fully consumed the food, completing the feeding process. The photos (except **D1** and **D6** when the larvae were not fed) were taken after the scheduled feeding.

A photographic sequence of preimaginal honey bee development based on the control group is shown in Figure 1.

**Figure 2:**
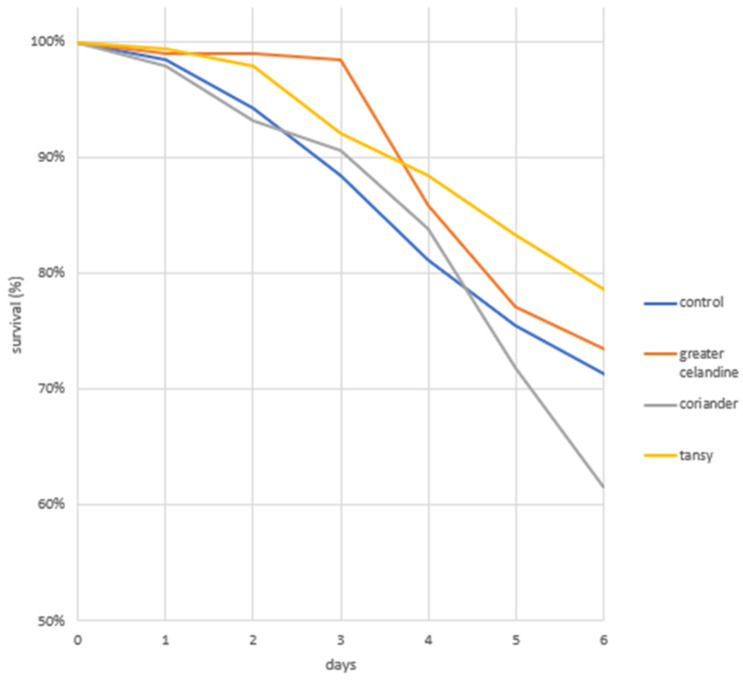
Percentage of survival of honey bee larvae reared in vitro and fed a control diet or diets containing plant infusions of coriander, greater celandine, and tansy.

The highest larval mortality was observed in the control group, with greater celandine and tansy on the third to fifth day after translocation. In the coriander group, the period of increased risk of mortality began on day 5 and persisted until the end of the experiment. A statistical analysis of larval survival by day among groups showed statistically significant changes from the second day after the transfer of larvae (D2) (Table 3). On the second day, the highest mortality rate was in the group fed with coriander (6.8%), the lowest mortality rate was in the group fed with the addition of greater celandine (1%), and this difference was not significant statistically. On day 3 (D3), the highest mortality was observed in the control group (11.5%) and this difference was significant statistically (*p*-value = 0.0009642). From the fourth day (D4) until the end of the experiment, a significantly higher survival rate in relation to the control group was characterized by the group fed with the addition of the tansy (*p*-value = 0.001). On the last day (D6), a statistically significant increase in larval mortality in the group fed with coriander infusion (from 28.1% to 38.5%, or by more than 10%) (*p*-value = 0.001) was observed. A similar difference was observed for the same group between D4 and D5. The survival rates (%) of all groups by day of life are shown in Figure 2.

## 4. Discussion

For many years, herbal plants have been used for various purposes, primarily in the pharmaceutical and food industries. They are an alternative to synthetic drugs both in unconventional medicine and in animal production [14]. So far, some natural remedies for treating honey bee infections have been tested. Propolis extracts and essential oils have been tested. Although fairly good results were obtained in all these studies, somewhat complicated procedures were used to obtain these extracts [15,16]. For this reason, in this study, we decided to investigate the activity of simpler aqueous extracts from three selected plant species. Active compounds contained in water infusions of coriander, tansy, and greater celandine were selected to study the effects on honey bee larvae of oral exposure to active substances, using three food compositions adapted to the age of the larvae. It was shown that the use of different food compositions (control diet and diets with the addition of plant infusions) in individual groups is a statistically significant factor affecting the probability of larval survival. The effects of nutritional supplements in the form of aqueous plant infusions on the survival of honey bee larvae have not yet been studied.

To date, in vitro rearing of larvae has mainly been used to study the effects of pesticide application on early life stages of the honey bee [13]. Due to the difficulty in rearing larvae in vitro, studies focusing on adult bee exposure to pesticides are more common than larval exposure [17]. Dai et al. (2017) conducted laboratory studies to determine the effects of the inclusion of selected pesticides in the diet of honey bee larvae, and the procedure they used to rear larvae is similar to that presented in this paper. The larvae were fed a larval diet adapted to their age (three compositions of the larval diet in vitro (A, B, C) administered on different days (D) were used. Diet A (D0–D1): royal jelly (44.25%), glucose (5.3%), fructose (5.3%), yeast extract (0.9%), and water (44.25%); diet B (D2): royal jelly (42.95%), glucose (6.4%), fructose (6.4%), yeast extract (1.3%), and water (42.95%); diet C (D3–D5): royal jelly (50%), glucose (9%), fructose (9%), yeast extract (2%), and water (30%).) or a basic diet containing various pesticides (exposure to the insecticide occurred on D3 at the time of diet C). The larvae were fed once a day (omitting the second day after postponement), receiving a total of 160 μL of diet. Mortality in the control group of 15% was achieved in the last days of larval life [17]. The composition of the diet, the method and time of administration, as well as the variability in the proportion of components depending on the day of life of each larva, were constantly tested [18]. In the experiment, the use of food compositions adapted to the age of the larvae (three different diets, A, B, and C) and the total amount of diet (160 μL) was supported by many previous studies conducted by various groups of scientists. The introduction of own modifications (in the form of the use of freeze-dried royal jelly and feeding twice a day with a reduced dose of the diet at designated intervals (±12 h)) in selected protocols of in vitro larval outflow did not result in a significant increase or decrease in larval survival compared to the experiments conducted by the groups of scientists described above.

The highest mortality obtained (38.5%) was for the group exposed to compounds contained in coriander, a plant widely used as a food additive and in phytotherapy worldwide. Despite the documented positive effects of coriander use on the human body, there are many reports of its limiting effects on the growth and survival of invertebrate organisms [19]. Active compounds in coriander have been shown to be toxic to plant pest species *Spodoptera littoralis* B. and *Tetranychus urticae* L., among others, effectively limiting population development both directly, through acute toxicity on egg stages, and indirectly, through delayed effects on immature and adult stages [20]. In a study on the toxicity of polyphenols contained in coriander on larvae of the greater hogweed (*Galleria mellonella* L.), a pest that feeds on beeswax, it was proven that exposure of larvae to the above-mentioned compounds resulted in high mortality, especially for a high dose (30 µL/mL), which led to 100% mortality within 4 days [11].

Increases in larvae survival rates were obtained at the level of 26.78% for tansy and 5.35% for greater celandine in comparison with the control. Surprisingly, however, both herb species are known for their adverse effects on insect organisms [21,22]. Much research has been carried out in order to prove the repellent or toxic action of compounds contained in the tansy, mainly on parasites of cultivated plants. An example of this is the experiment performed on two parasitic species, *Pieris rapae* L. and *Plutella xylostella* L., pests of cabbage plants. In both laboratory and field tests, a reduction in caterpillar feeding on cabbage leaf discs after the use of a water extract of tansy was confirmed. The larvae reared on cabbage leaves treated with the extract of wolfsbane took longer to develop to the pupa stage and the pupae weighed less than individuals reared on control leaves [21]. The limiting effect of tansy was also confirmed on caterpillars of the species *Trichoplusia ni* [22]. On the other hand, studies using crude extracts of greater celandine have shown its significant insecticidal activity against some lepidopteran forest pests such as *Lymantria dispar* L., *Clostera anastomosis* L., and *Dendrolimus superans* B. [23]. Despite these reports, in the present study no limiting or toxic effects of the active compounds of wolfsbane and bindweed on honey bee larvae were observed.

## 5. Conclusions

This research has shown that selected plant extracts influence honey bee larvae in laboratory conditions. Therefore, when assessing the toxicity of preparations, it is important to check their effects on all developmental stages, not only on adults. Plant extracts intended to be used in beekeeping should also be tested on all developmental stages of honey bees, from the moment of translating the larvae to the completion of the development of workers, under laboratory conditions.

## Figures and Tables

**Table 1:** Amount of each dietary component per 100 larvae for control group (with distilled water).

Component of a Diet	Diet A		Diet B		Diet C	
	[g]	%	[g]	%	[g]	%
Glucose	0.133	5	0.160	6	1.125	9
Fructose	0.133	5	0.160	6	1.125	9
Yeast extract	0.023	1	0.033	1	0.250	2
Distilled water	1.846	74	1.792	72	7.917	63
Royal jelly	0.367	15	0.356	14	2.083	17
Sum	2.500	100	2.500	100	12.500	100

**Table 2:** Daily food volumes and the diet used (A, B, or C), supplied to the larvae depending on the age of the larvae.

Day	Diet	Volume of the Diet (μL)
D0	A	20
D1	-	-
D2	B	2 × 10
D3	C	2 × 15
D4	C	2 × 20
D5	C	2 × 25
	SUM	160

**Table 3:** Mortality of honey bee larvae fed with three different food compositions (diets A, B, and C), adjusted to the age of larvae and treated with biologically active compounds contained in aqueous infusions of herbs of selected plant species; survival assessment consisted of daily counts of dead larvae; N = number of surviving larvae (total of 192 larvae for each group; 4 repetitions of 48 larvae).

		Control		Coriander *C. Sativum*		Greater Celandine *C. Majus*		Tansy *T. Vulgare*	
Day	Diet	N	% Mortality	N	% Mortality	N	% Mortality	N	% Mortality
D0	A	192 ^A^	0%	192 ^A^	0%	192 ^A^	0%	192 ^A^	0%
D1	-	189 ^A^	1.6%	188 ^A^	2.1%	190 ^A^	1%	191 ^A^	0.5%
D2	B	181 ^A,B^	5.7%	179 ^A^	6.8%	190 ^B^	1%	188 ^A,B^	2.1%
D3	C	170 ^A^	11.5%	174 ^A^	9.4%	189 ^B^	1.6%	177 ^A^	7.8%
D4	C	156 ^A^	18.7%	161 ^A,B^	16.1%	165 ^A,B^	4.1%	170 ^B^	11.5%
D5	C	145 ^A^	24.5%	138 ^A^	28.1%	148 ^A^	22.9%	160 ^B^	16.7%
D6	-	137 ^A^	28.6%	118 ^B^	38.5%	141 ^A,C^	26.6%	151 ^C^	21.4%

## Data Availability

The datasets generated and/or analyzed during the current study are available from the corresponding author on reasonable request.
